# The Impact of Symmetry: Explaining Contradictory Results Concerning Working Memory, Reasoning, and Complex Problem Solving

**DOI:** 10.3390/jintelligence5020022

**Published:** 2017-05-18

**Authors:** Alexandra Zech, Markus Bühner, Stephan Kröner, Moritz Heene, Sven Hilbert

**Affiliations:** 1 Institut für Notfallmedizin und Medizinmanagement, Klinikum der Universität München, LMU München, 80336 Munich, Germany; 2 Department of Psychology, Ludwig-Maximilians-University Munich, 80802 Munich, Germany; buehner@psy.lmu.de (M.B.); heene@psy.lmu.de (M.H.); 3 Department of Education, Friedrich-Alexander-University Erlangen-Nuremberg, 90478 Nuremberg, Germany; stephan.kroener@fau.de; 4 Faculty of Psychology, Educational Science, and Sport Science, University of Regensburg, 93053 Regensburg, Germany; sven.hilbert@ur.de

**Keywords:** symmetry, content, working memory, reasoning, complex problem solving, MultiFlux

## Abstract

Findings of studies on the unique effects of reasoning and working memory regarding complex problem solving are inconsistent. To find out if these inconsistencies are due to a lack of symmetry between the studies, we reconsidered the findings of three published studies on this issue, which resulted in conflicting conclusions regarding the inter-relations between reasoning, working memory, and complex problem solving. This was achieved by analysing so far unpublished problem solving data from the study of Bühner, Krumm, Ziegler, and Plücken (2006) (*N*= 124). One of the three published studies indicated unique effects of working memory and reasoning on complex problem solving using aggregated scores, a second study found no unique contribution of working memory using only figural scores, and a third study reported a unique influence only for reasoning using only numerical scores. Our data featured an evaluation of differences across content facets and levels of aggregation of the working memory scores. Path models showed that the results of the first study could not be replicated using content aggregated scores; the results of the second study could be replicated if only figural scores were used, and the results of the third study could be obtained by using only numerical scores. For verbal content, none of the published results could be replicated. This leads to the assumption that not only symmetry is an issue when correlating non-symmetrical data, but that content also has to be taken into account when comparing different studies on the same topic.

## 1. Introduction

Symmetry in the amount of aggregation is a concept that has a long history in scientific research across many fields (see [1]). It was Egon Brunswik [2] who famously adopted the concept into psychology using his lens model 2 (as cited by Wittmann and Süß [1]). The main proposition of this concept is that psychological constructs differ regarding their level of aggregation (or generalization) and that this has an impact on the interrelations between different constructs (see [3]). It is suggested that two constructs need to be on a comparable level of generalization (i.e., be “symmetrical”) to obtain a less biased empirical correlation. For example, Wittmann and Süss [1] could show that, when the criterion comprises a lower level of generalization, prediction is better with predictors from that same level than with aggregated predictors, even though reliability might be higher for the aggregated predictor. For example, in this study [1], spatial working memory capacity could not be predicted as well by *g*-factor reasoning as by the spatial category of reasoning. 

Apart from the issue of symmetry within one study (i.e., predictor and criterion have to be on the same level of generalization to obtain less biased results), there is a second aspect of symmetry that should not be neglected. Symmetry is also important when comparing different studies with each other. As different levels of symmetry between studies can lead to different conclusions regarding correlations between constructs being measured on different levels of aggregation across studies, the results of studies investigating the same cognitive constructs on differing levels of symmetry are prone to be contradictory—even if the constructs were measured symmetrically within each study. This aspect of symmetry is central for the present article, and examples for studies using different levels of aggregation are therefore described in detail further below.

Referring to the aspect of symmetry between studies, Wittmann [4] introduced the symmetry principle as a “necessary prerequisite for successful validation“. He argued that, if the constructs in one study are measured on a more generalized level than in another study, those studies are not comparable. The aim of this study is to empirically investigate how issues regarding both aspects of symmetry may explain contradictory results in the literature concerning the relation between working memory, reasoning, and complex problem solving.

For answering this question, we utilized the data from the study of Bühner, Krumm, Ziegler, and Plücken [5] and compared the results to those of Wittmann and Süss [1], Bühner, Kröner, and Ziegler [6], and Greiff, Krkovic, and Hautamäki [7]. Note, however, that the present analyses of the Bühner et al. [5] data are novel, as the previous publication based on these data did not include the results of the problem solving scenario, due to a different research focus. The fact that different reasoning and working memory tests were used in all of these studies enabled us to take a look at the same question on different levels of aggregation.

### 1.1. Symmetry between Different Studies

The general problem of non-replicability in psychology has recently been broadly discussed and has raised serious questions about the soundness of psychological results [8,9,10], and findings about the relationship between working memory and reasoning might not be an exception, given the contradictory results that are mentioned in Section 1.2. A possible lack of symmetry—within or between studies—is, however, rarely mentioned in the literature. Rather, it is often just vaguely described under the more general term “conceptual replications”, that is, a study based on an original study uses different measures to assess the effect under consideration for a notable exception (see [11]). To achieve unbiased estimates of correlations within one study and to compare results between different studies, it is important that the constructs are measured on corresponding levels of generalization within each study and that the studies being compared operate on the same level of generalization.

The level of generalization often coincides with a certain hierarchical position of a construct in a theoretical model. For example, in the Cattell–Horn–Carroll theory of cognitive abilities (see [12]), three levels (“strata”) of cognitive are assumed. The first stratum is depicted by so-called narrow abilities such as deductive reasoning or memory span. On the second stratum, broad abilities such as fluid reasoning (Gf) and short-term memory reside. They are usually measured as an aggregate of different subtests. At the apex of the model, general intelligence (“*g*”) represents the aggregate of the stratum II abilities. In this case, aggregation not only leads to a reduction of task-specific variance, but, at the same time, creates factors that are on a higher level of generalization and are therefore better suited to predict criteria that are on a higher level of generalization as well. This illustrates that, when judging the level of generalization of an aggregated score, it is crucial to consider the heterogeneity of contents among its constituents. For example, a score that has been derived from three different verbal tasks would be on a lower level of generalization than a score derived from one verbal, one numerical and one figural task. Of course, a score derived from several verbal, numerical, and figural tasks would be at an even higher level of generalization as it measures both the construct on a broad level and at the same time controls for task-specific variance. Regarding scenarios measuring complex problem solving, things are slightly different. Due to their complex nature, even single scenarios will involve abilities from different areas. Thus, even an aggregated score of several runs of a single complex problem solving scenario may result in problem solving scores that are on a level of generalization comparable to fluid reasoning [13]. Of course, using several scenarios might nevertheless be the preferable solution [7]. This is in line with the results reported by Wittmann and Hattrup [11] who also extensively discussed the importance of symmetry in the analysis of complex tasks.

### 1.2. Contradictory Results on the Relation between Working Memory, Reasoning, and Complex Problem Solving

We aimed at investigating the impact of symmetry both within and between studies on the issue of the relation between working memory, reasoning, and complex problem solving. While there may be other studies on the relationship of the three constructs under scrutiny (e.g., [14,15]), especially regarding their construct validity (see [16]), we focused on the studies of Witmann and Süss [1] who applied several older scenarios with known psychometric issues for measuring complex problem solving, Bühner et al. [5] who applied MultiFlux, a scenario that aimed at overcoming the most severe reliability and validity issues, and Greiff et al. [7] who applied MicroDYN, which is one of the most recent developments in the realm of complex dynamic systems, featuring multiple exploration trials and rational item construction. Thus, we selected studies that dealt with the relationship of working memory, reasoning, and complex problem solving in quite different ways and came to quite different conclusions.

While Wittmann and Süss [1] as well as Greiff et al. [7] found unique effects for both reasoning and working memory when explaining variance in complex problem solving, Bühner et al. [6] found no evidence for unique effects of reasoning above and beyond working memory.

These three studies differ concerning the level of generalization regarding the measurements of the constructs. Wittmann and Süss [1] used content aggregated scores of working memory, reasoning, and complex problem solving that were based on several different tasks, respectively. On the other hand, Bühner et al. [6] used an aggregate of several figural tasks to measure reasoning and working memory and several runs of one single scenario to measure complex problem solving. In one version of their models, Greiff et al. [7] used numerical working memory tasks and reasoning tasks and measured complex problem solving as an aggregate of different scenarios within MicroDYN.

To test the hypotheses that the seemingly contradictory results of these three studies are to be explained by the different levels of symmetry within studies as well as varying levels of generalization between studies, we reanalysed so far unpublished data on complex problem solving that had been collected in the context of the study by Bühner et al. [5]. While in these data the criterion—complex problem solving—was measured using several runs of one simulation only, these data allowed us to estimate this criterion by working memory and reasoning on different levels of symmetry. We were able to vary symmetry by assessing both working memory and reasoning in four different ways by selecting different subsets from several figural, verbal and numerical tasks: (1) using all tasks for content aggregated scores; (2) selecting only figural tasks for figural scores; (3) selecting only numerical tasks for numerical scores; and (4) selecting only verbal tasks for verbal scores. Thus, our model (2) was comparable to Bühner et al. [6] who also used several runs of one simulation for measuring complex problem solving as well as several figural tasks to measure reasoning and working memory. Our model (3) came as close as possible to Greiff et al. [7] as—in one version of their models—they had the same approach in general using only numerical tasks to measure working memory and reasoning and applied several different MicroDYN scenarios for complex problem solving. Regarding level of generalization, the measurement for complex problem solving used by Greiff et al. [7] is on a slightly higher level than MultiFlux, since their measure is an aggregation of nine different MicroDYN tasks, while, in our model, only four MultiFlux simulations were conducted that only differed regarding the underlying model. As the MicroDYN tasks at least follow the same rationale, we argue that the models are at least comparable.

Our model 1 is as close as possible to that of Wittmann and Süss [6]. However, they operated on a completely symmetrical level as they measured both complex problem solving as well as intelligence and working memory with an aggregate of several different tasks. Even if the aggregate for complex problem solving is not balanced regarding its demands, it is still on a higher level of generalization than MultiFlux as—in contrast to the four MultiFlux simulations—Wittmann and Süss used three tasks with very different rationales. Since in our model (1) complex problem solving was measured with several trials of the same task, these studies are not symmetrical to each other.

With regard to symmetry between studies, it is thus to be expected that our model (2) can replicate those of Bühner et al. [6] and we find results similar Greiff et al. in our model (3) [7]. On the other hand, we expect different results than Wittmann and Süss [1] for our model (1). This is due to the fact that the model of Wittmann and Süss is symmetrical (predictors and criterion are on the same level of generalization) while our model (1) is not (aggregated working memory and reasoning are on a higher level of generalization than complex problem solving as measured with one task only). As there is currently no comparable research on the prediction of verbal working memory and reasoning tasks on complex problem solving, we make no specific assumptions for our model (4).

### 1.3. The Relation between Working Memory and Reasoning

To make research on the specific contributions of working memory and reasoning regarding complex problem solving a meaningful venture, it is of utmost importance that both constructs are not merely different labels for the same construct. There is a wealth of studies concerning the relation between reasoning and working memory. Many of them report high correlations between the two constructs (e.g., [17,18,19,20]). In these studies, several conjectured reasons for these high correlations can be found, including the fact that measures for both constructs require participants to use short-term memory for an overview (see [21]) and central executive components [22,23]. Although these results led some researchers to the assumption that reasoning and working memory are, in fact, different labels for the same construct [18,24,25,26], other researchers come to a different conclusion c.f. [21]. They point out the fact that, although the correlations are often very high, they are seldom perfect, even after correcting for measurement error. For example, the meta-analysis of Ackerman et al. [21] resulted in a mean estimated true score correlation of only .47, and even the studies showing the strongest correlations between working memory and reasoning did not provide evidence for more than 50 percent of shared variance between those constructs [27]. This, in turn, led to a multifaceted debate regarding the statistical procedures for the meta-analysis and the strength of the relationship as well as its adequate interpretation [27,28], eventually leading to the conclusion that the picture of results indicates two (highly) correlated but distinct constructs. 

### 1.4. Complex Problem Solving

“Complex problem solving takes place for reducing the barrier between a given state and an intended goal state with the help of cognitive activities and behavior” [29] (p. 682). It differs from simple problem solving as exact features of the problem are unknown at the beginning and may change over time [29]. In contrast to reasoning, complex problem solving requires interaction with the task environment in order to gain knowledge about the system [30].

Since the distribution of personal computers in the past decades, a widespread operationalization of complex problem solving is performance in computer-simulated scenarios. These were constructed to represent complex real-world scenarios, for example being the mayor of a small village or managing a tailor shop [11,31]. Nevertheless, with early computer simulations, correlations of complex problem solving scores with related psychological constructs seemed to be low [31]. This could later be attributed to design flaws and the unsatisfying reliability of the early simulations [13,32]. After constructing scenarios that took these issues into account, the complex problem solving scores derived from them correlated moderately to large with other cognitive abilities [33,34]. Furthermore, more recent investigations showed that a broader operationalization of intelligence results in higher correlations with complex problem solving and left the latter without any incremental variance shared with complex criteria of academic success [16,35], thereby further highlighting the importance of the symmetry principle. In addition, the number of complex problem solving scenarios used in scientific studies has been growing during the past years [36]. As stated above, in this study, we analyzed data from Bühner et al. [5] including thus far unpublished data regarding complex problem solving from MultiFlux, an abstract task with clearly specified goals as well as highly reliable scores for the knowledge regarding the simulation model and its application in simulation-related tests that are independent of domain-specific prior knowledge. Note that the task has been modified for the present study to include multiple simulation runs and to provide a more efficient way of assessing simulation knowledge based on an automatic evaluation of causal diagrams (“causal knowledge”) that replaces the rule “knowledge tasks” used by Kröner et al. [13]. Further details on these measures are presented in Section 2. 

### 1.5. Hypotheses

Under the assumption that the seemingly contradictory results of Bühner et al. [6], Wittmann and Süss [1], and Greiff et al. [7] could be explained by lacking symmetry between the studies and by the differing contents that were used, we expected to be able to replicate the results of the three studies used for comparison to a differing degree. This leads to the following four hypotheses: (1) effects of reasoning and working memory on complex problem solving regarding MultiFlux differ depending on the content category used; (2) the findings of Bühner et al. [6], namely, that figural reasoning does not predict complex problem solving as measured with MultiFlux above and beyond figural working memory, can be replicated when using figural predictors; (3) results similar to the findings of Greiff et al. [7]—namely, that numerical working memory does not predict complex problem solving above and beyond numerical reasoning—can be found when using numerical predictors. We do not expect a direct replication, as Greiff et al. used a different measurement for complex problem solving with different task-specific demands; and (4) when aggregated scores are used for measuring working memory and reasoning but not complex problem solving, the findings of Wittmann and Süss [1], with reasoning and working memory being distinguishable predictors of complex problem solving, will not be found in that extent.

## 2. Materials and Methods

To answer our research questions, we reanalysed the data of a previous study [5] and included results concerning complex problem solving that were not used in the original publication.

### 2.1. Participants

The sample consisted of *n* = 124 undergraduate psychology students—three students more than in the subsample of Bühner et al. [5] because some variables for which missing values had led to the exclusion of participants were irrelevant for the present study. The mean age was 21.7 years (*SD* = 3.2, range = 18–46), and 80.5 percent of the participants were female. Due to the recruiting at the office of admission and enrolment services, almost all (*n* = 115) of the subjects were studying in their first semester. The participants received course credits and feedback on their test results.

### 2.2. Instruments and Procedure

The tests were administered in three sessions of about three hours each. The Intelligence-Structure-Test 2000-R (Intelligenz-Struktur-Test 2000-R, IST [37]) was conducted during the first session alongside two attention tests that were of no interest for this study. The participants worked on the computer simulation MultiFlux [33] as a measurement for complex problem solving during the second and on the working memory test battery [38] during the third session. All tests were conducted on personal computers in a laboratory and with a maximum of five participants per session. For a detailed description of test measures, see Bühner et al. [5].

#### 2.2.1. Multiflux

**Figure 1:**
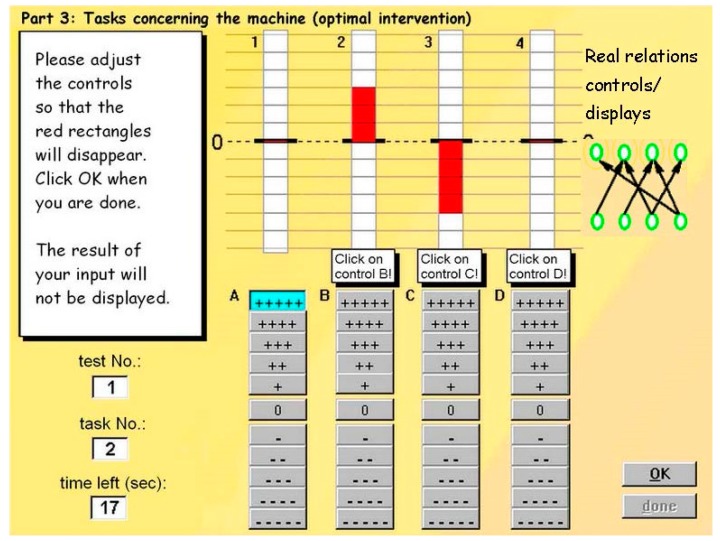
The MultiFlux machine.

The scenario “MultiFlux” [33] was used to measure complex problem solving. MultiFlux is a computer simulation of a fictitious machine with four controls and four displays that are related to each other in a complex manner (Figure 1). All relations between controls and displays were of equal strength. Setting a control to “+” caused an increase of one unit in all displays connected to this control, setting it to “++” lead to an increase of two units, and so on. Participants first have to explore the simulation. During the exploration phase, they have to experiment with the simulation to identify relationships between controls and displays, which they will draw into a causal diagram that remains on screen during further exploration trials. At the end of the exploration phase, they are presented with the correct causal diagram and work on a simulation-based test with rule application items. For these items, the displays of the simulation show deviations from the optimal (zero) level. It is the participants’ task to find the pattern of the controls that would make the displays hit the zero position in the next step (which is actually not presented to prevent violations of local stochastical independence of the test items).

Compared to Kröner et al. [13]**,** an improved version was applied that already contained one of the features that Greiff and Funke [39] would also implement in their Minimal Complex Systems approach: (1) the participants did not only explore one single simulation, but rather repeatedly explored several versions of MultiFlux; (2) the simulations differed in the underlying model to adapt difficulty to the university student population; and (3) for the same reason, the rule knowledge items used in previous studies [8,33,34] have been replaced by causal knowledge scores that were assessed from a causal diagram that could be drawn on screen by the participants during work on the simulation. Causal knowledge represents the sum of correctly identified existing relations plus the correctly non-identified non-existing relations between controls and displays at the end of each exploration phase, summed up across all four simulation runs. Thus, causal knowledge scores represent the cognitive process of knowledge acquisition, as described by Greiff, Wüstenberg, Holt, Goldhammer, and Funke [40]. Moreover, rule application scores were assessed via simulation-based tests as described in the literature, thus representing knowledge application [40]. These scores represent the sum of all 16 dichotomously scored rule-application items (four from each simulation run). Knowledge acquisition and knowledge application have been found to be predictors of academic success above and beyond reasoning compare [40], but this finding has been questioned by recent studies that applied a broader operationalization of intelligence, as described above [16,35].

The simulation consisted of two phases: the exploration phase and the intervention phase. During the exploration phase, the relations between controls and displays of the fictitious machine (the simulation model) had to be identified in a maximum number of four steps by adjusting the controls and observing the effects on the instruments. In each of the four exploration steps, these effects could be observed as soon as participants had confirmed that all instruments had been finally adjusted. From these observations, participants might conclude which controls were related to which instruments and might draw arrows in a structural diagram accordingly. From the structural diagram resulting from the exploration phase, causal knowledge (CausalKnow) was computed as the sum of correctly identified existing and non-existing relations between controls and displays.

For answering each of four items in the intervention phase, participants were provided with the correct simulation model as displayed in the upper right corner of Figure 1. For each item, the display was set to a certain position and participants had to adjust the controls so that all the displays reach the zero position, which had been defined as an optimum. The rule-application scores (RuleApp) were calculated dichotomously. Participants received a point for each rule-application task that they solved completely correctly during the intervention phase.

To increase both reliability and time-efficiency of the simulation as compared to that used by Bühner et al. [6] and Kröner et al. [13], some changes were applied to the procedure: firstly, participants had to deal with four simulation runs in which different models of increasing complexity had to be explored and four accompanying intervention tasks had to be solved, respectively, as described above. CausalKnow and RuleApp scores were then computed as aggregates of scores from all four simulation runs. To enable this, the rule knowledge items used by Kröner et al. [13] and Bühner et al. [6] were replaced by an analysis of the arrows drawn in the structural diagram. Both RuleApp and CausalKnow were used as measurements of complex problem solving in all path analyses conducted in this study.

#### 2.2.2. I-S-T 2000-R

The subtests of the basic module of the Intelligence-Structure-Test 2000-R (Intelligenz-Struktur-Test 2000-R, IST [37]) were conducted. This test is a well validated and frequently used intelligence test in Germany. It assesses reasoning in the figural, numerical, and verbal content categories with three subtests each, resulting in nine subtests. For each subtest, the number of correct tasks was assessed and summed up for the three subtests making up one category. For the verbal models, the subtests’ sentence completion, analogies and similarities were used (referred to as REAS_V). The numerical facet (referred to as REAS_N) was measured using the tasks’ arithmetic problems, sequences of numbers and arithmetic operators. In addition, figural reasoning (referred to as REAS_F) was measured via selecting figures, tasks with three-dimensional dice and matrices. The reasoning score used in the aggregated models (referred to as REAS) was calculated as the sum of all previously stated tasks.

#### 2.2.3. Working Memory Tests

The working memory scores were measured using a shortened version of the test battery programmed by Oberauer et al. [38], based on their working memory model. This model consists of two facets, operation and content, comprising three operation categories and the two content categories (numerical/verbal and figural/spatial), respectively. The operation categories are supervision, storage in the context of processing and relational integration. An aggregate of the three operation categories was used in the analyses.

For the assessment of supervision, switching tasks were used. In the verbal condition, participants had to decide whether a word had one or two syllables (if it appeared in one of the upper two cells) or whether a word was a plant or an animal (if it appeared in one of the lower two cells), respectively. Because the stimulus cell changed clockwise, the participants always had to use the same criterion twice and then switch to the other criterion. In addition, tasks with numerical (odd vs. even; 500 vs. 500) and figural (one connected vs. two separate patterns; symmetrical vs. non-symmetrical pattern) content were used. General switching costs were calculated as the difference of the log-transformed reaction times from no-switching trials and the corresponding baseline choice reaction tasks [38].

To assess storage in the context of processing, the choice reaction tasks that had already been used for the supervision tasks were combined with short-term memory tasks from the same content domain. First, the material that had to be memorized was presented. Afterwards, the choice reaction tasks had to be solved for five seconds. For the figural scores, the pattern task as described by Oberauer et al. [38] was used. The score used in the current study was derived from the number of correctly remembered items [38].

Relational integration was measured using monitoring tasks [38]. Participants had to observe words (verbal condition) or numbers (numerical condition) in a 4 × 4 matrix that were interchanged randomly. The task was to press the ‘space’ key when a certain pattern was visible. For the verbal and numerical condition, this was the case when three rhyming words or, respectively, three numbers with the same final digit formed a horizontal, vertical or diagonal line on the screen. No memory capacity was needed because all items were visible on the screen the whole time. The score was calculated by subtracting the number of false reactions from the number of correct reactions. For the figural condition, the flight control task was used [38]. Planes were represented by small triangles that moved across the screen with different speeds and in different directions. In addition, there were mountains on the screen that were represented by brown squares. The participants’ task was to keep the planes from colliding with each other or with the mountains. They could do so by stopping and redirecting the planes with the mouse. The participants had a limited credit that shrunk with every stop of the traffic and with every crash. Each item took 12 s without pause times. The score used for further calculations was the mean number of collisions per round. They were inverted to facilitate interpretation. 

### 2.3. Statistical Analysis

#### 2.3.1. Path Analysis

We conducted path analyses with AMOS 22 (IBM SPSS, Chicago, IL, USA) using maximum-likelihood estimations. All coefficients reported are based on standardized solutions. Cut-off values of the Root Mean Square Error of Approximation (*RMSEA*) ≤ .06 and the Standardized Root Mean Square Residual (*SRMR)* ≤ .08 were applied to assess the global-fit between the tested model and the data [41]. Additionally, the Comparative-Fit-Index (*CFI*) was inspected. According to Hu and Bentler [41], a cut-off value of .95 indicates appropriate global-fit. In addition, the χ^2^-model test was used for model evaluation. 

#### 2.3.2. Models

Our models tested the interrelations between the categories of working memory, reasoning, and complex problem solving with aggregated scores, comprising three content categories (model 1). In addition, separate models for figural (model 2), numerical (model 3), and verbal (model 4) content were tested. In version (a) of the four models, REAS predicts CausalKnow and RuleApp. Analogous to Kröner et al. [13] and Greiff et al. [7], CausalKnow was specified as a predictor for RuleApp as well. In version (b) of the models, paths from WM to CausalKnow and RuleApp were added to test the incremental predictive power that working memory could provide above and beyond reasoning. We also calculated achieved power [42,43] to detect misspecified models assuming an RMSEA of .06 in the population against the alternative of RMSEA = .08.

## 3. Results

Table 1 depicts the correlations between the facets of complex problem solving, working memory, and reasoning. In Table 2, sample size, means, standard deviations, and reliability indices of the test scores are provided. As we were not able to get hold of the original raw scores of the data, the reliability scores for reasoning stem from the original publication of the data [5]. For the problem solving and working memory scores, we were able to calculate McDonald’s ω total, which is reported in Table 2. McDonald’s ω total gives a reliability estimate of the overall variance in the data that is due to a general factor and specific factors. ω hierarchical is a reliability estimate for the variance that is due to the general factor only [44].

Model fit statistics for all path analysis are provided in Table 3. Model (a) differs from model (b) to the effect that, in model (a), no direct effects of working memory scores on complex problem solving are specified. Model fit statistics for model (b) are not provided since this model has 0 degrees of freedom and is thus saturated. 

### 3.1. Model 1 (Aggregated Predictors)

**Figure 2:**
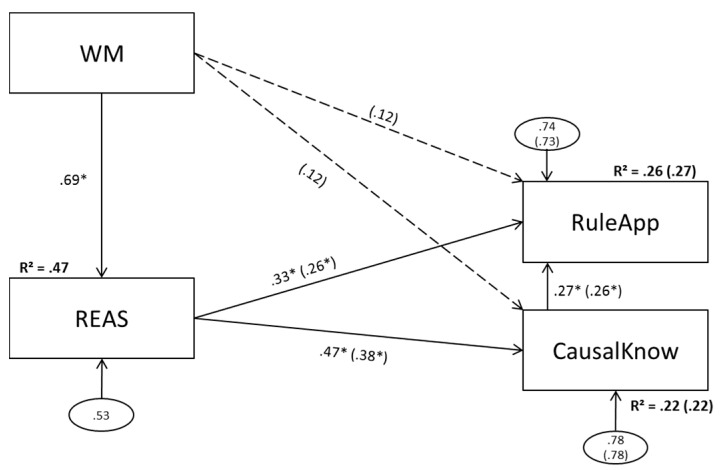
Model 1a(b): Aggregated working memory components as predictors of reasoning; reasoning (and working memory) as predictors of problem solving. WM = working memory; REAS = reasoning; CausalKnow: = causal knowledge; RuleApp = rule application. Dashed lines and values in parentheses depict changes in model 1b. Statistically significant paths are marked with an asterisk (α = .05).

Model 1 is depicted in Figure 2.

#### 3.1.1. Model 1a (No Direct Effect of Working Memory Scores on Complex Problem Solving)

The data did not deviate significantly from the model (χ^2^ [2] = 2.30, *p* = .32) and the global model fit was acceptable. The paths from REAS on RuleApp and CausalKnow reached statistical significance. REAS explained 22 percent of CausalKnow variance and 26 percent of RuleApp variance.

#### 3.1.2. Model 1b (Direct Effects of Working Memory Scores on Complex Problem Solving)

The two paths added from WM to the complex problem solving scores did not reach statistical significance. The paths from REAS to CausalKnow and RuleApp remained significant. Explained variance in the complex problem solving scores could not be improved (.26 vs. .27 and .22 vs. .22).

### 3.2. Model 2 (Figural Predictors)

**Figure 3:**
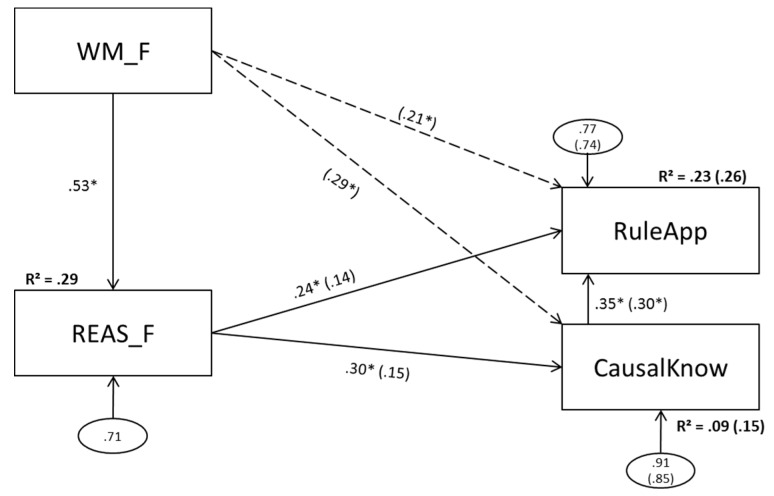
Model 2a(b): Figural working memory components as predictors of figural reasoning; figural reasoning (and figural working memory scores) as predictors of problem solving. WM_F = figural working memory; REAS_F = figural reasoning; CausalKnow = causal knowledge; RuleApp = rule application. Dashed lines and values in parentheses depict changes in model 2b. Statistically significant paths are marked with an asterisk (α = .05).

Model 2 is depicted in Figure 3.

#### 3.2.1. Model 2a (No Direct Effect of Figural Working Memory Scores on Complex Problem Solving)

The data deviated significantly from the model and the global model fit was not acceptable. Because parameter estimates from misspecified models can be seriously biased [45], leading to incorrect conclusions, we do not interpret parameter estimates and multiple R^2^ of miss-fitting models.

#### 3.2.2. Model 2b (Direct Effects of Figural Working Memory Scores on Complex Problem Solving)

The two added paths from WM_F to CausalKnow and RuleApp reached statistical significance. REAS_F, in turn, did not explain incremental variance of CausalKnow and RuleApp above and beyond working memory. Explained variance in CausalKnow and RuleApp improved to 15 and 26 percent, respectively.

### 3.3. Model 3 (Numerical Predictors)

**Figure 4:**
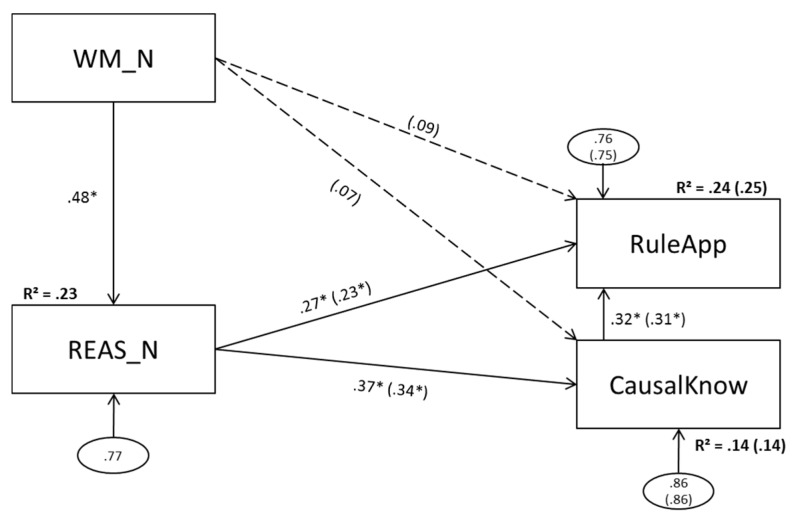
Model 3a(b): Numerical working memory components as predictors of numerical reasoning; numerical reasoning (and numerical working memory scores) as predictors of problem solving. WM_N = numerical working memory; REAS_N = numerical reasoning; CausalKnow = causal knowledge; RuleApp = rule application. Dashed lines and values in parentheses depict changes in model 3b. Statistically significant paths are marked with an asterisk (α = .05).

Model 3 is depicted in Figure 4.

#### 3.3.1. Model 2a (No Direct Effect of Numerical Working Memory Scores on Complex Problem Solving)

Model 3a revealed an acceptable global model-fit and did not deviate significantly from the data. All paths in this model reached statistical significance. Fourteen percent of CausalKnow and 24 percent of RuleApp variance could be explained.

#### 3.3.2. Model 3b (Direct Effects of Numerical Working Memory Scores on Complex Problem Solving)

Both paths added from WM_N to CausalKnow and RuleApp missed statistical significance while the paths from REAS_N to CausalKnow and RuleApp remained significant. Explained variance did not increase (.24 vs. .25 and .14 vs. .14).

### 3.4. Model 4 (Verbal Predictors)

**Figure 5:**
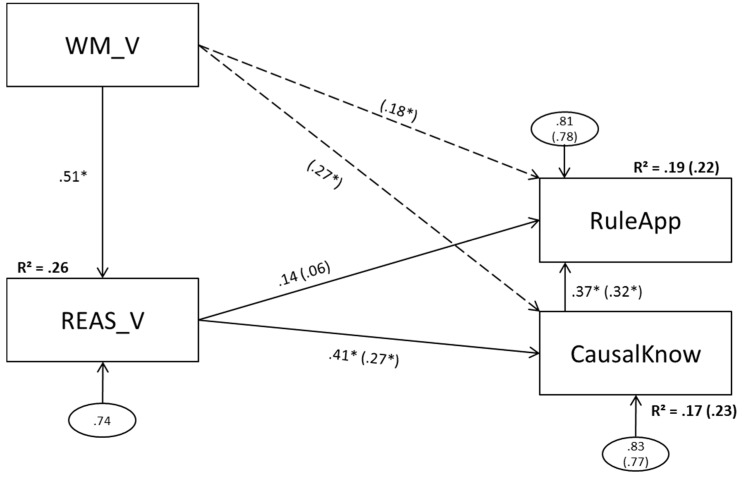
Model 4a(b): Verbal working memory components as predictors of figural reasoning; verbal reasoning (and verbal working memory) as predictors of problem solving. WM_V = verbal working memory; REAS_V =verbal reasoning; CausalKnow = causal knowledge; RuleApp = rule application. Dashed lines and values in parentheses depict changes in model 4b. Statistically significant paths are marked with an asterisk (α = .05).

Model 4 is depicted in Figure 5.

#### 3.4.1. Model 4a (No Direct Effect of Verbal Working Memory Scores on Complex Problem Solving)

The global fit of model 4a (see Figure 5) did not meet the criteria and the data deviated significantly from the model. We therefore refrain from interpreting the parameter estimates and multiple *R*^2^ of this non-fitting model.

#### 3.4.2. Model 4b (Direct Effects of Verbal Working Memory Scores on Complex Problem Solving)

The added paths from WM_V to CausalKnow and RuleApp reached statistical significance. The path coefficient from REAS_V to RuleApp stayed insignificant, whereas the coefficient from REAS_V to CausalKnow decreased but remained significant. Twenty-three percent of CausalKnow variance and 22 percent of RuleApp variance could be explained.

## 4. Discussion

### 4.1. Main Results

The present data reconcile the seemingly contradictory findings of Wittmann and Süss [1], Bühner et al. [6], and Greiff et al. [7] and suggest differences in symmetry levels as well as working memory and reasoning content between the studies as presumable reasons for the discrepancies. The study shows that the explanatory power of reasoning and working memory regarding complex problem solving as measured with MultiFlux varies depending on the level of aggregation and the content. In line with Bühner et al. [6], figural reasoning did not display a unique effect above and beyond working memory on any of the complex problem solving measures used (see Figure 3). For numerical content, in line with Greiff et al. [7], the exact opposite was the case (see Figure 4): numerical reasoning predicted complex problem solving significantly while the numerical working memory components had no additional effect for the prediction of MultiFlux complex problem solving. The verbal model (see Figure 5) differentiated between causal knowledge and rule application. While, for causal knowledge both verbal working memory and verbal reasoning had significant effects, for rule application, verbal reasoning had no significant predictive power. As expected, the results of Wittmann and Süss [1] could not be replicated when using content aggregated scores for reasoning and working memory, as Wittmann and Süss [1] used a score for complex problem solving that was aggregated as well, while, in this study, complex problem solving was measured by one task only. Since Wittmann and Süss used three tasks that are very different, this leads to a higher level of generalization (even if the demands are not balanced for content). Since, in our model, a complex problem was measured using four versions of the MultiFlux task that all have the same rationale, the degree of task specific variance in this study is higher. This results in lacking symmetry between both studies.

While, in the figural models, figural working memory was the only significant predictor of complex problem solving when controlled for figural reasoning, in the numerical models, it was the other way around. One might suspect that this finding could be related to differences in the reliabilities of reasoning and working memory. However, these reliabilities were very similar in three of the four models, making it possible to rule out distortions due to differential attenuation as an explanation for these findings. Another possible explanation might be related to specific demands of the MultiFlux tasks. In this particular simulation, participants might have made arithmetic operations requiring numerical reasoning, while, at the same time, having to translate their results into visual representations, thus requiring figural working memory. Since the operation has to be conducted in one step, we suspect that this task does not require unique contributions of figural reasoning, even though the bivariate correlations proved to be significant. This does not hold for numerical and verbal reasoning, since they can both be processed verbally and processed in a more sequential manner see [46]. Since Greiff et al. [7] used MicroDYN, a measurement for complex problem solving that allows participants to make their decisions in several steps (as opposed to the one step approach used in MultiFlux), we would hypothesize that with MicroDYN—despite a representation combined of figural and numerical aspects that is similar to MultiFlux—figural working memory would not be a predictor for complex problem solving above and beyond reasoning.

Thus, it would be interesting to replicate the study by Bühner et al. [6] using various simulations including MicroDYN as operationalisations of complex problem solving and differentiating between different contents used for the measurement of working memory and reasoning, as it was done in this study. Moreover, a larger sample size would be preferable to be able to better compare differences in effect sizes across the various content facets. These results are discussed in detail in the subsequent paragraphs.

### 4.2. Symmetry as a Central Factor for the Comparability of Different Studies

The obtained results clearly show that symmetry within studies plays a central role in the prediction of MultiFlux complex problem solving scores, as Wittmann [4] has suggested. Aggregating scores from different categories completely altered the implied relationships and lead to different models. As Wittmann and Süss describe in their 1999 article [1], it can be a problem if two correlating abilities are not measured comparably broadly [1]. In our case, symmetry in the aggregated model was lower than in the content-specific models, since the criterion, complex problem solving, had not been measured as broadly as the aggregated reasoning and working memory scores. Thus, in the content-specific models, where both the predictors and the criterion were measured using only one task for every construct, symmetry was higher than in the aggregated model, which compensated for the lower reliability of the working memory measures as compared to the aggregated model when explaining criterion variance. Still, the present study might not have met perfectly the criteria of symmetry as outlined by Wittmann and Süss [1] due to the broader operationalization of reasoning and working memory compared to complex problem solving. This topic is further discussed in the Section 4.4.

Aside from the symmetry problems within the models used in this study, symmetry between studies, i.e., similarity of aggregation levels, may be considered to be an important factor for the comparison of different studies as well. The results of Bühner et al. [6] and Greiff et al. [7] cannot be compared to those of Wittmann and Süss [1], even though the designs and the applied model are comparable to each other, because they are not on the same level of aggregation.

### 4.3. Implications for the Discriminant Validity of Reasoning and Working Memory

Many earlier studies showed substantial effects of reasoning on complex problem solving [1,6,7,11,13,47]. In line with these findings, reasoning was a statistically significant predictor in all models where paths from working memory to causal knowledge were not estimated. Some of the studies cited above did not control for working memory—and for those that did, an inconsistent pattern of results was observed. The present study, however, shows that when predicting performance in complex problem solving scenarios, models drawing on content aggregated scores for reasoning and working memory may lead to different findings than models drawing on content-specific scores. One reason for such effects is the variation of relative importance of working memory and reasoning across content factors, depending on the scenario—or group of scenarios—under scrutiny. In the present investigation, this variation has been demonstrated for the complex problem solving test MultiFlux. This is an important result as it enabled us to provide support for differential discriminant validity of working memory and reasoning (see [27,28] for an exhaustive discussion on this topic) on a higher level of aggregation. It shows that studies operating on a lower level of the hierarchical construct, whether symmetrically or asymmetrically, may draw a biased picture due to the influence of task-specific variance. If one wants to draw conclusions about the relationships between actual (broad) constructs, a high level of aggregation and high symmetry between the construct levels (as argued by Süss and Wittmann [1] and Wittmann and Hattrup [11]) seems strongly warranted. This is in line with previous research addressing symmetry issues, for example on the relation between intelligence and academic success, e.g., [48]. However, further research needs to analyse if such evidence can also be found in other criteria within and without the realm of cognition. 

### 4.4. Limitations and Perspectives for Further Research

A reanalysis of our findings with a different criterion measure for complex problem solving would be interesting. The scenario MultiFlux [13] used by Bühner et al. [6], like most problem solving scenarios, has not been constructed with the aim of balanced task requirements across content categories. Therefore, it might be that the relation between MultiFlux complex problem solving, reasoning, and working memory differs depending on the content used to measure working memory or reasoning. Nevertheless, in prior studies, MultiFlux showed quite strong correlations with reasoning measures aggregated across all three content factors that are very improbable to be totally substituted by aggregated measures of working memory [13]. As MultiFlux may have different demands than MicroDYN, our model (3) is not a replication of Greiff et al. [7]. In addition, for a replication of Wittmann and Süss [1], it would be necessary to use an aggregated score of different tasks as a measurement for complex problem solving to archive symmetry within the study as well as between the studies. Further studies should also investigate potential effects of different operationalisations of causal knowledge (as quality of causal diagrams drawn vs. scores in rule knowledge items requiring the prediction of a simulation input-vector on a given simulation state) on correlations between causal knowledge and rule application scores. The latter turned out to be rather low in the present study—probably as an effect of less method variance shared by the operationalisations that were used in the present study.

Moreover, Oberauer et al. [38,49] considered verbal and numerical working memory content as a common category, which is divided in the present study, while reasoning is commonly divided into three content categories, e.g., [50], as it was also done here. The strong connection of verbal and numerical stimuli may have had a strong impact in this study, since the numerical MultiFlux stimuli may be well be processed verbally, as discussed above (see also [46]). This may have also led to the finding that—even though MultiFlux requires processing of numerical rather than verbal stimuli—model 4 showed strong associations between CPS and verbal working memory as well as verbal reasoning. This assumption is further supported by the results of Hilbert, Schwaighofer, Zech, Sarubin, Arendasy, and Bühner [51], who found that training with verbal working memory tasks was associated with superior performance in numerical working memory tasks and vice versa, while no such connection was found for figural working memory tasks. Even though the correlational pattern may therefore be biased, increasing the symmetry at least regarding the content facet has still shown its impact and, as mentioned above, even the distinction between verbal and numerical working memory content revealed interesting results.

Finally, we emphasize the need for direct replication studies using larger samples as in the present study. As the power analysis on the achieved power for the RMSEA revealed [43], achieved power for each model was very low, implying that the probability to detect misfitting models was below the minimum required level of .80 [42]. Furthermore, the second version of the models was saturated and thus their model fit could not be tested. The results and interpretations of the models and their parameters must therefore be regarded as preliminary.

## 5. Conclusions

Taken together, symmetry turned out to be an important factor, not only when comparing correlations within a single study but also for the comparison between different studies on the same topic. Thus, as suggested by Wittmann and Süss [1] as well as Wittmann and Hattrup [11], the level of aggregation seems to be decisive for the conclusions that may be drawn from an investigation of cognitive constructs. Task-specific contents are prone to bias a given result, depending on the content and the given level of aggregation of all constructs in question. In this sense, the present investigation demonstrated how the replication of various studies could or could not be achieved, depending not only on different contents on the same level of aggregation but also aggregation of the content for at least two of the constructs in question and further showed that the replication of the study conducted by Wittmann and Süss [1] could not be achieved, likely due to a lack of an aggregated score for complex problem solving. This leads to the conclusion that, if one wants to explore the relation between cognitive abilities, and not merely between different tasks or tests, aggregation across different material categories is indispensable.

## Figures and Tables

**Table 1:** Correlations between problem solving, working memory, and reasoning. Significant correlations are marked in bold typefaces (*α* = .05).

Tests Scores	1	2	3	4	5	6	7	8	9
**Problem Solving**									
(1) Causal Knowledge									
(2) Rule Application	**.42**								
**Working Memory**									
(3) Working Memory	**.39**	**.39**							
(4) Working Memory (Figural)	**.37**	**.40**	**.90**						
(5) Working Memory (Numerical)	**.23**	**.28**	**.76**	**.56**					
(6) Working Memory (Verbal)	**.41**	**.35**	**.83**	**.61**	**.53**				
**Reasoning**									
(7) Reasoning	**.47**	**.46**	**.69**	**.66**	**.48**	**.58**			
(8) Figural Reasoning	**.30**	**.35**	**.51**	**.53**	**.38**	**.33**	**.79**		
(9) Numerical Reasoning	**.37**	**.39**	**.62**	**.59**	**.48**	**.50**	**.86**	**.52**	
(10) Verbal Reasoning	**.41**	**.29**	**.41**	**.35**	**.18**	**.52**	**.58**	**.21**	**.32**

**Table 2:** Means, range, standard deviations, sample sizes, and reliabilities of the raw test scores.

Tests Scores	*M* (Range)	*SD*	*n*	*r_tt_*
**Problem Solving**				
Causal Knowledge	53.16 (26–64)	9.17	124	ω = .80 ^b^
Rule Application	5.86 (0–16)	4.37	124	ω = .72 ^b^
**Working memory**				
Working Memory	1.14 (0.23–1.75)	0.27	124	ω = .81 ^b^
Working Memory (Figural)	0.59 (−0.53–1.19)	0.32	124	ω = .83 ^b^
Working Memory (Numerical)	2.17 (1.38–2.82)	0.28	124	ω = .31 ^b^
Working Memory (Verbal)	1.54 (−0.11–2.30)	0.37	124	ω = .45 ^b^
**Reasoning**				
Reasoning	114.47 * (73–154)	18.58	124	ω = .93 ^a^
Reasoning (Figural)	33.75 * (14–55)	8.48	124	α = .88 ^a^
Reasoning (Numerical)	40.08 * (21–58)	9.77	124	α = .94 ^a^
Reasoning (Verbal)	40.64 * (11–52)	5.92	124	α = .74 ^a^

a = Cronbach’s alpha; b = McDonald’s ω total; * = equivalent to average scores concerning age and education.

**Table 3:** Model fit statistics for path analysis. Significant *p*-values are marked in bold typefaces (α = .05).

Tests Scores	χ^2^ [*df*]	*p*	RMSEA [CI90]	Achieved Power RMSEA ^a^	CFI	SRMR
Model 1a (aggregated)	2.30 [2]	.317	.035 [.000; .187]	.09	.998	.031
Model 2a (figural)	13.10 [2]	**.001**	.213 [.114; .330]	.09	.879	.094
Model 3a (numerical)	1.62 [2]	.443	.000 [.000; .169]	.09	.999	.033
Model 4a (verbal)	11.88 [2]	**.003**	.201 [.102; .318]	.09	.893	.089

RMSEA = Root Mean Square Error of Approximation, CFI = Comparative Fit Index, SRMR = Standardized Root Mean Square Residual; ^a^ = for H_0_: RMSEA ≤ .06 against H_1_: RMSEA = .08, with α = .05.
